# Gastrointestinal Tract Challenges: Chronic Inflammatory Fibroid Polyps Demystified

**DOI:** 10.7759/cureus.49068

**Published:** 2023-11-19

**Authors:** Abeer Qasim, Jasbir Makker

**Affiliations:** 1 Internal Medicine, BronxCare Health System, New York, USA; 2 Gastroenterology, BronxCare Health System, New York, USA

**Keywords:** chronic inflammatory cells, gastrointestinal endoscopy, inflammatory fibroid polyps, benign gastrointestinal tumors, vanek tumors

## Abstract

An inflammatory fibroid polyp (IFP), also known as Vanek’s tumor, is an uncommon benign tumor typically found as a solitary, intraluminal polyp in the gastrointestinal (GI) tract. Chronic IFP is characterized by persistent or recurrent inflammatory features, distinct histopathological findings, and a potential for significant GI tract involvement. Typically, IFPs occur predominantly in the gastric antrum, small intestines, and recto-sigmoid colon. They initiate within the submucosal layer and extend into the lamina propria, resulting in a noticeable bulging of the mucosal layer. They may breach the mucosal barrier on rare occasions, leading to ulceration and bleeding. This ongoing bleeding can induce persistent blood loss and symptoms typical of hypovolemic shock. When of smaller size, these growths might be accidentally detected during an endoscopic examination. Conversely, if the lesions are sizable, they can prompt symptoms of obstruction like queasiness, retching, and abdominal discomfort. Here, we present a case of a 47-year-old female who underwent a screening colonoscopy and was found to have an IFP.

## Introduction

Vanek's tumor, also known as inflammatory fibroid polyp (IFP), ranks among the rarest benign tumors in the small bowel [1]. IFP was initially characterized by Vanek in 1949 as a submucosal granuloma with infiltration of eosinophils [2]. This anomaly is defined by the growth of extensively vascularized fibrous tissue and infiltration by varying quantities of diverse inflammatory cells. The exact cause of this condition remains unidentified [3]. The highest occurrence is typically observed in individuals between their sixties and seventies, with a slight male predominance [4]. They are usually asymptomatic; however, if symptomatic, the main symptom is abdominal pain when the polyp is situated in the stomach. In the small bowel, it can result in intussusception or intestinal obstruction. Less common symptoms encompass vomiting, loose stools, bloody stools, tenesmus, and altered bowel habits [5]. The primary treatment for most cases involves the endoscopic removal of the lesion. There have been no reported instances of recurrence or complications specific to IFPs [6]. Here, we present an atypical occurrence of a chronic IFP in a patient with long-standing constipation. The condition was detected as part of a regular colonoscopy screening.

## Case presentation

**Figure 1:**
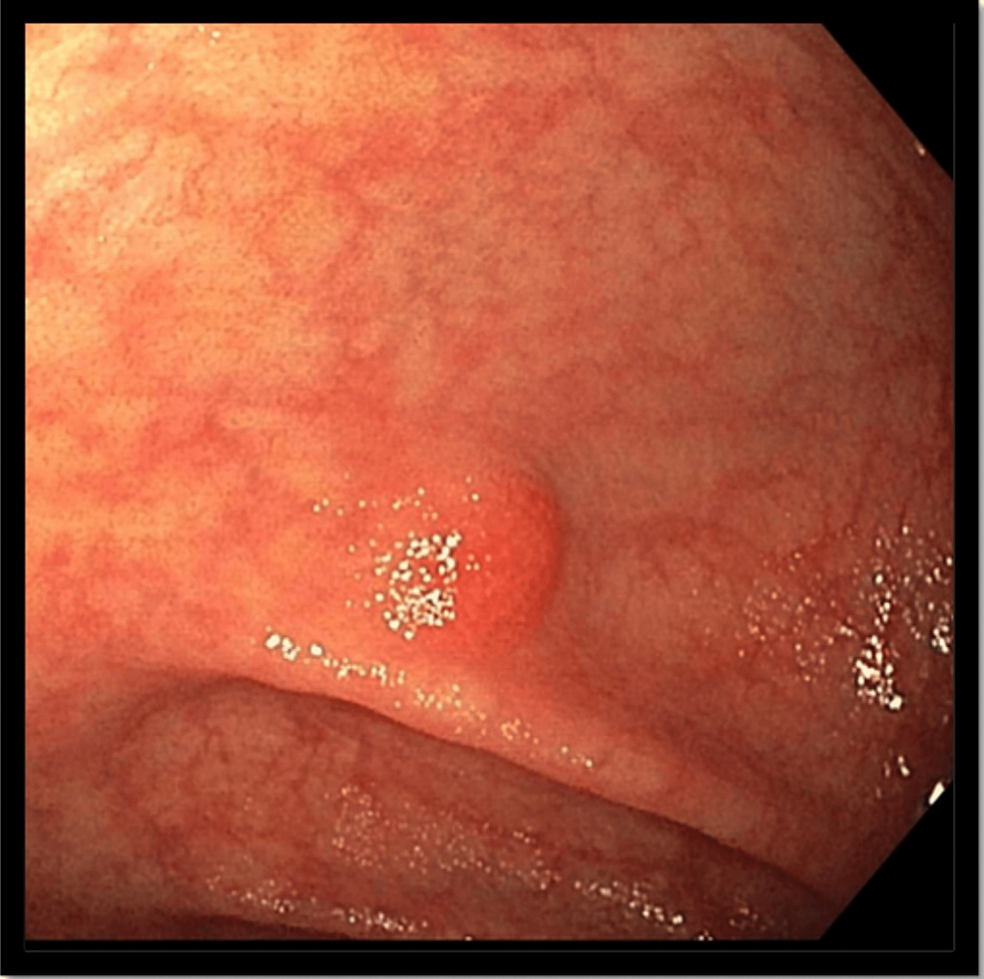
Sessile polyp (12 mm) at the hepatic flexure.

A 47-year-old female with no medical history visited the clinic for a routine colonoscopy screening. The patient had a complaint of chronic constipation, characterized by infrequent bowel movements occurring only once a week; however, she denied any nausea, vomiting, diarrhea, abdominal pain, dysphagia, loss of appetite, early satiety, or unintentional weight loss. Also, she had no history of hematemesis, melena, or hematochezia. She never had any prior workup, including endoscopy and colonoscopy. The patient denied any smoking or using illicit drugs, prior surgical history, or any use of any medications at home. Family history was not significant for gastrointestinal (GI) malignancy. When she arrived at the clinic, her vitals were stable, with a blood pressure of 109/74 mmHg, a pulse of 69 beats per minute, and a body temperature of 97.9 degrees Fahrenheit. The physical examination was unremarkable. The patient was informed about the procedure's risks versus benefits, and she agreed to have the colonoscopy after giving her informed consent. The colonoscopy revealed a 12 mm sessile polyp at the hepatic flexure (Figure 1) and asymptomatic non-bleeding external and internal hemorrhoids, and polypectomy was done. 

**Figure 2:**
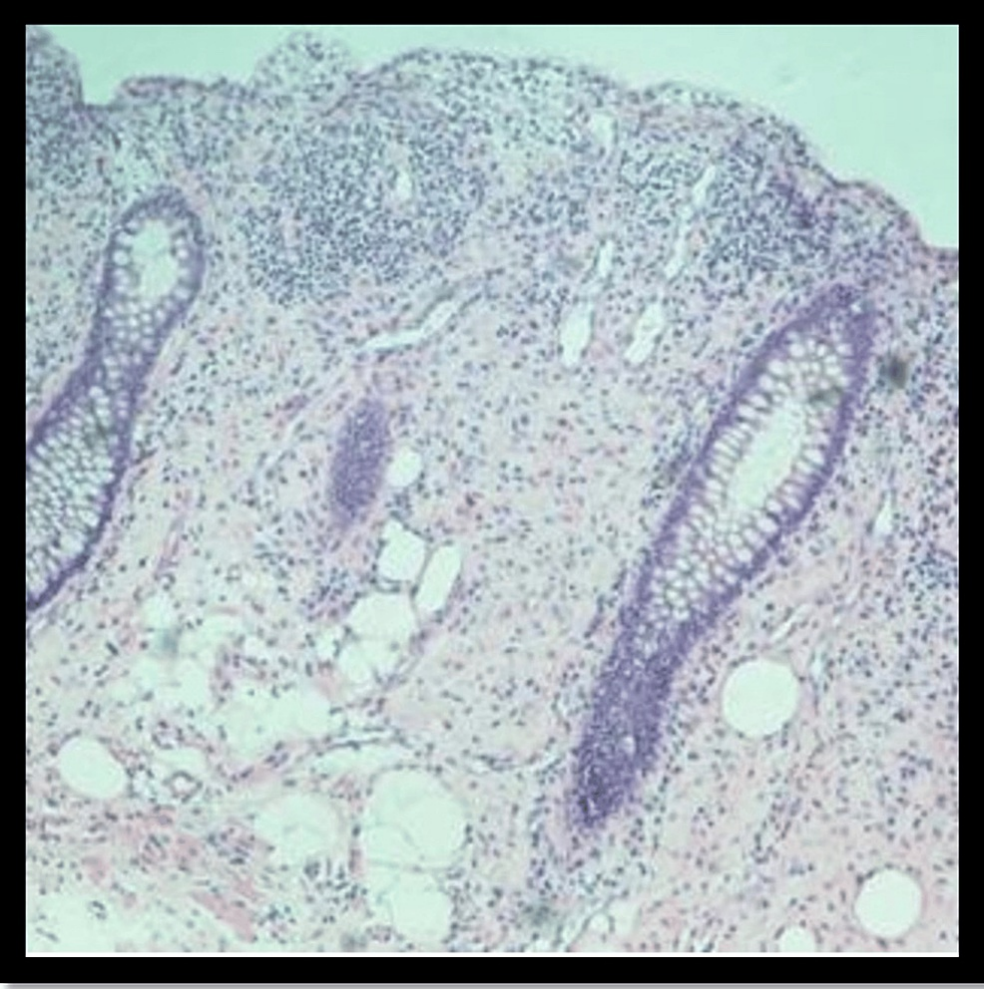
Enlarged fragments of colonic mucosa.

The histopathological analysis of the colon polyp showed that the fragments of colonic mucosa and submucosa were enlarged due to the growth of fibrous tissue with scattered groups of eosinophils (Figure 2).

**Figure 3:**
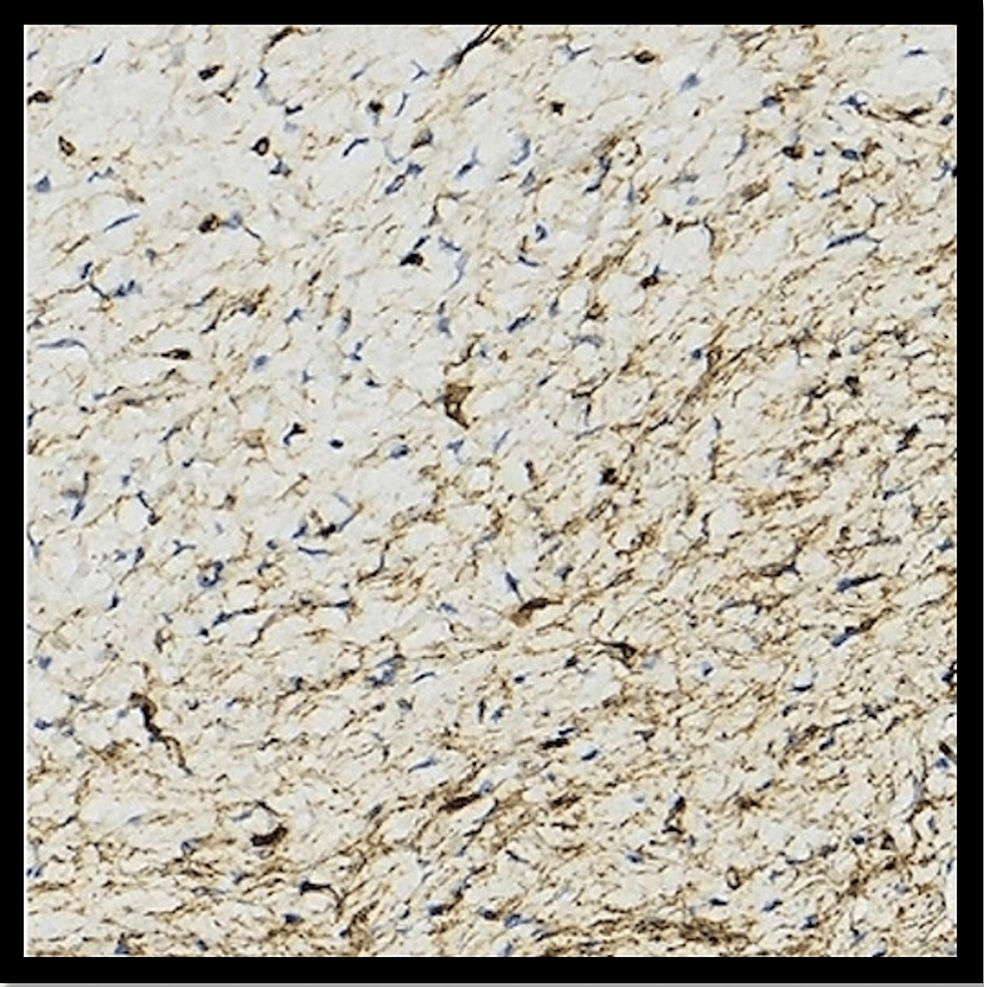
Immunostaining positive for CD34.

Immunostaining was conducted, revealing that the affected tissue had widespread positivity for CD34 and was negative for S100, epithelial membrane antigen (EMA), and glucose transporter-1 (GLUT-1), consistent with an IFP (Figure 3). S100-positive monophasic synovial sarcoma may enter in differential diagnosis with GI clear cell sarcomas (CCS) and CCS-like tumors of the GI tract.

The patient was discharged and was advised to undergo a surveillance colonoscopy in three years. 

## Discussion

IFP is an uncommon benign solitary growth originating from the submucosal layer of the GI tract. IFP typically manifests at various locations within the GI tract: approximately 66-75% of cases appear in the gastric antrum, 18-20% in the small intestine, and 4-7% in the large intestine. About 1% of occurrences are observed in the duodenum, esophagus, and gallbladder, with less than 1% involving the appendix [2,3]. Notably, the ileal segment is the primary site where these polyps often lead to intussusception [7]. The approximate occurrence of IFP within the broader population ranges from 0.3% to 0.5% [8]. Historically, it was believed to be a reactive condition linked to an autoimmune or an allergic response. However, identifying heightened expression and mutations in the platelet-derived growth factor receptor alpha (PDGFRA) has shifted the understanding of IFP towards being seen as a neoplastic process [9]. PDGFRA is situated on chromosome 4q12 and activates pathways that stimulate cellular growth signals and influence cell differentiation [9]. Mutations in this gene have been detected in a sizable proportion of IFPs, ranging from 21.7% to 69.6%. These mutations are predominantly located in exon 12 (more prevalent in cases originating in the small intestine, often involving deletion or insertion) or exons 14 or 18 [10]. It rarely occurs in a familial syndromic setting (Devon polyposis syndrome) [11]. 

IFPs typically do not show symptoms and are usually detected incidentally during endoscopy or laparotomy. However, if they do cause symptoms, the clinical manifestation is primarily influenced by the size and where they are located anatomically. In the stomach, prevalent symptoms often include vomiting, epigastric pain, and bleeding. Intussusception and obstruction are typical symptoms of the lesion in the small bowel. Our patient was asymptomatic. In a macroscopic view, IFP typically appears as a single fibrous mass with a rounded or oval shape, exhibiting a grayish or yellowish-gray coloration. It often shows ulcers on the surface of the mucosa [12], whereas, under light microscopy, variations in collagen fiber quantity and the ratio of infiltrating cells can indicate a developmental shift, where a densely populated lesion with a scarcity of collagen signifies a more recent evolution in the condition along with the growth consisted of many blood vessels, varying in thickness, and an inflammatory infiltration within a fibrous tissue, containing a significant number of eosinophilic white blood cells [13]. Macroscopically the differential diagnosis should include GI stromal tumors (GISTs), leiomyomas, inflammatory myofibroblast tumors, or leiomyosarcomas. The histological findings of our patient showed that the fragments of colonic mucosa and submucosa were enlarged due to the growth of fibrous tissue with scattered groups of eosinophils. Conversely, colonic lesions commonly present with symptoms such as colicky pain, weight loss, diarrhea, bleeding, and anemia [14].

Typically, the diagnosis of IFP is confirmed through histological and immunohistochemical analysis of the surgical or endoscopic specimen. To confirm the diagnosis of IFPs, immunohistochemical staining is essential. IFPs typically display positive staining for CD34 and vimentin. In some cases, they may also show positive staining for smooth muscle actin (SMA), calponin, CD35, and cyclin D1 [15]. Approximately 10-20% of cases display partial reactivity for SMA and desmin. Conversely, C-KIT (CD117, tyrosine-protein kinase), discovered on GIST-1 (DOG1), and S-100 consistently show negative results. A recent study involving 14 IFP cases revealed that three of them did not exhibit a response to CD34. Among the CD34-positive cases, concentric stromal proliferations (CP) were observed. The researchers inferred that IFP cases with CP might have a distinct origin or development compared to those without CP. They further proposed that IFP cases featuring CP could potentially stem from a specific subgroup of interstitial dendritic cells [16]. The immunostaining for our patient was positive for CD34 and was negative for S100, EMA, and GLUT-1. During upper GI endoscopy, esophagogastric and duodenal IFPs appear as raised lesions within the wall, presenting a smooth surface that is frequently ulcerated. Colorectal lesions are detected through colonoscopy, while small-bowel lesions are typically identified during surgical procedures, often due to obstructive symptoms [17]. Our patient underwent a routine colonoscopy and was found to have IFP. In the case of intussusception, an abdominal CT scan is the imaging of choice. 

Regarding treatment, most IFPs can be removed through endoscopic resection, and surgery is seldom required. IFPs that originate below the Treitz ligament may cause an acute abdomen, often due to intussusception. In such cases, exploratory laparoscopy or laparotomy is typically advised as the optimal approach for treating intussusception attributed to IFP [18]. To date, there is only one documented case of a recurring lesion, and there hasn't been any indication of malignant behavior [19]. Our patient’s polyp was removed by polypectomy. Following that she was advised for a repeat colonoscopy in three years for surveillance. 

## Conclusions

IFP continues to be an uncommon noncancerous tumor within the GI tract, predominantly found in the gastric antrum and the rectosigmoid colon. While its exact cause is not fully comprehended, it is widely recognized as a benign neoplasm primarily driven by PDGFRA. Diagnosing an IFP can pose challenges, given its often nonspecific clinical symptoms. Biopsy samples from endoscopic procedures may not always yield sufficient information for a histological diagnosis. The most reliable diagnosis is achieved through immunohistochemistry analysis of the removed tumor. 
